# Scoliosis Research Society members attitudes towards physical therapy and physiotherapeutic scoliosis specific exercises for adolescent idiopathic scoliosis

**DOI:** 10.1186/s13013-015-0041-z

**Published:** 2015-05-27

**Authors:** Cindy L. Marti, Steven D. Glassman, Patrick T. Knott, Leah Y. Carreon, Michael T. Hresko

**Affiliations:** Spinal Dynamics of Wisconsin, 2300 North Mayfair Road, Suite 555, Wauwatosa, WI 53226 USA; Department of Orthopaedic Surgery, University of Louisville School of Medicine, 550 S. Jackson St., 1st Floor ACB, Louisville, 40202 Kentucky USA; Norton Leatherman Spine Center, 210 East Gray Street, Suite 900, Louisville, 40202 Kentucky USA; Rosalind Franklin University of Medicine and Science, 3333 Green Bay Road, North Chicago, IL 60064 USA; Department of Orthopaedic Surgery, Children Hospital Boston, Harvard Medical School, Boston, MA 02115 USA

**Keywords:** Adolescent idiopathic scoliosis, Scoliosis specific exercise, Survey

## Abstract

**Background:**

Attitudes regarding non-operative treatment for adolescent idiopathic scoliosis (AIS) may be changing with the publication of BRAiST. Physiotherapeutic Scoliosis Specific Exercises (PSSE) are used to treat AIS, but high-quality evidence is limited. The purpose of this study is to assess the attitudes of members of the Scoliosis Research Society towards PSSE.

**Methods:**

A survey was sent to all SRS members with questions on use of Physical Therapy (PT) and PSSE for AIS.

**Results:**

The majority of the 263 respondents were from North America (175, 67 %), followed by Asia (37, 14 %) and Europe (36, 14 %). The majority of respondents (166, 63 %) prescribed neither PT nor PSSE, 28 (11 %) prescribed both PT and PSSE, 39 (15 %) prescribe PT only and 30 (11 %) prescribe PSSE only. PT was prescribed by 67 respondents, as an adjunct to bracing (39) and in small curves (32); with goals to improve aesthetics (27) and post-operative outcomes (25). Of the 196 who do not prescribe PT, the main reasons were lack of evidence (149) and the perception that PT had no value (112).

PSSE was prescribed by 58 respondents. The most common indication was as an adjunct to bracing (49) or small curves (41); with goals to improve aesthetics (36), prevent curve progression (35) and improve quality of life (31). Of the respondents who do not prescribe PSSE, the main reasons were lack of supporting research (149), a perception that PSSE had no value (108), and lack of access (63). Most respondents state that evidence of efficacy may increase the role of PSSE, with 85 % (223 of 263) favoring funding PSSE studies by the SRS.

**Conclusion:**

The results show that 22 % of the respondents use PSSE for AIS, skepticism remains regarding the benefit of PSSE for AIS. Support for SRS funded research suggests belief that there is potential benefit from PSSE and the best way to assess that potential is through evidence development.

## Background

Physiotherapeutic Scoliosis Specific Exercise (PSSE) has been advocated as a potentially valuable tool in the treatment of adolescent idiopathic scoliosis (AIS) [1–11]. Postulated treatment effects include preventing curve progression, minimizing respiratory dysfunction, preventing spinal pain syndromes, and improving aesthetics via postural correction [1–11]. These treatment effects may lead to avoidance of brace treatment or surgery [12, 13]. PSSE is also commonly used in conjunction with brace treatment, and is suggested to increase the efficacy of bracing [14–16]. In general, the role of PSSE is more widely accepted in Europe versus North America.

The evidence-base supporting PSSE treatment regimen is very limited, as most of the published literature consists of case studies and small cohort studies. A systematic review in 2012 identified only 12 relevant studies and described the evidence as poor quality because all were at level IV or lower in terms of their Level of Evidence [6, 9]. This deficiency is due in part to inconsistent indications for PSSE treatment, the diversity of therapy programs, ranging from outpatient postural training to intensive and prolonged in-patient regimens, and the difficulty in assuring compliance when patients are receiving therapy over an extended period. The best available literature, is a recent published randomized clinical trial of 110 patients that demonstrates PSSE to be superior to standard physical therapy (PT) in patients older than 10 years with small AIS curves (10° to 25°) and Risser sign <2 [5].

Despite limited high quality evidence, the possibility of effective nonsurgical treatment for AIS is obviously attractive. Although U.S. surgeons have historically been skeptical regarding the role of these conservative exercise treatment options, attitudes may be changing. Publication of the BRAiST study, which demonstrated efficacy of bracing for AIS based upon clearly defined clinical parameters [17], has encouraged both patients and surgeons to the potential for brace treatment. At least in the public sector, this interest seems to extend beyond bracing to encompass a wider array of nonsurgical options.

The purpose of this study is to explore the attitudes of scoliosis surgeons regarding Physiotherapeutic Scoliosis Specific Exercise (PSSE). Beyond questions of awareness and acceptance, we sought to determine whether surgeons believe that evidence development regarding PSSE was an important research priority.

## Methods

After receiving Institutional Board Approval, a web-based survey was sent to all 1200 Active and Candidate members of the Scoliosis Research Society. The survey (Table 1) included questions regarding membership status, training and years in practice. For the respondents who prescribe standard PT or PSSE, their indications and goals for standard PT and PSSE were queried. For those who do not prescribe standard PT or PSSE, they were further queried on why they do not prescribe standard PT or PSSE. In addition, respondents were asked if they have observed an increased interest in PSSE, what evidence of this they have observed, and if they anticipate an increased role for PSSE in the future. All analysis was performed using PASW Statistics GradPack 17.0 (Chicago, IL: SPSS Inc.). Fisher’s exact test was used to determine difference in responses between regions of practice, years in training and type of training.

**Table 1:** Survey questions and responses

Question	Response
Member type	Active
	Candidate
Country	
Birth date	
Please describe your training.	Orthopedic Spine Surgeon
	Pediatric Orthopedic Surgeon
	Neurosurgical Spine Surgeon
	Other (please specify)
How many years have you been in practice?	0-10 years
	11-20 years
	>21 years
Do you treat AIS in your practice?	Yes/No
Do you refer AIS patients for STANDARD physical therapy?	Yes/No
Which patients? (check all that apply)	Small curves – not braced
	Curves in braces
	Pre-surgical
	Post-surgical
	Only for families who request therapy
	Patients with symptoms/pain
Indicate primary goals of referring for STANDARD physical therapy? (check all that apply)	Prevent curve progression
	Improve aesthetics via postural correction
	Prevent or treat spinal pain
	Prevent or treat respirary dysfunction
	Prevent surgery
	Improve post-surgical outcome with pre-op PT
	Postpone surgery
Why don’t you refer? (check all that apply)	Lack of perceived value in standard PT
	Lack of access
	Lack of patient interest/compliance
	Lack of perceived value in standard PT
	Lack of research to support standard PT
	Cost to patient
Do you refer patients for SCOLIOSIS SPECIFIC EXERCISE?	Yes/No
Which methods? (check all that apply):	Schroth
	Lyon Method
	Side Shift Method
	Functional Individual Therapy of Scoliosis
	Scientific Exercise Approach to Scoliosis
Which patients? (check all that apply)	Small curves – not braced
	Curves in braces
	Improved posture/Aesthetics
	Post-surgical
	Pre-surgical
	Only for families who ask
What are your goals for SCOLIOSIS SPECIFIC EXERCISE (SSE)? (check all that apply)	Improved posture/Aesthetics
	Improving surgical outcome
	Postponing surgery
	Preventing Curve Progression
	Preventing surgery
	Improved posture/Aesthetics
Why don’t you refer? (check all that apply)	Lack of access to SSE trained therapists
	Lack of research to support SSE
	Lack of perceived value in SSE
	Lack of access to SSE trained therapists
	Lack of patient interest/compliance
Have you observed an increased interest in SCOLIOSIS SPECIFIC EXERCISE?	Yes/No
In what ways do you see evidence of this change? (check all that apply)	Hospitals and Clinics in your area are promoting SSE
	More research/publications about PT or SSE
	Patients and families are asking for PT or SSE
	Doctors are referring more frequently to PT or SSE
	Hospitals and Clinics in your area are promoting SSE
Why not? (check all that apply)	Lack of patients/family interest in SSE
	Lack of physician education or interest about benefit of SSE
	Lack of research/publications about of SSE
	Hospitals and Clinics in your area do not provide SSE
Do you anticipate an increased role for SSE in the future?	Yes/No
What would facilitate this change?	
Do you support the use of SRS research funds to support higher quality research regarding the potential benefit of SSE for AIS?	Yes/No
Please add any additional perspectives you would like to share:	

## Results

There were a total of 266 respondents (22 %). Three were excluded from the analysis as they indicated that that they did not treat AIS patients, leaving 263 respondent surveys available for analysis. The majority of respondents (175, 67 %) were from North America, with an almost equal number from Asia (37, 14 %) and Europe (36, 14 %), a small number of surgeons from South America and the Middle East.

Almost all the respondents were Orthopedic Surgeons; 159 (60 %) categorized their spinal deformity training primarily as Orthopedic Spine and 95 (36 %) as Pediatric Orthopedics. Clinical experience, measured by years in practice, was almost equally represented, with most (110, 42 %) in practice for more than 20 years, 81 (31 %) have been in practice between 11 and 20 years and 69 (26 %) have been in practice for ten years or less.

The majority of respondents (166, 63 %) prescribed neither PT nor PSSE, 28 (11 %) prescribed both PT and PSSE, 39 (15 %) prescribe PT only and 30 (11 %) prescribe PSSE only. Sixty-seven (25 %) surgeons prescribe standard PT for their patients with AIS. Although the reported use of standard PT was more common in Europe, there was no statistically significant difference in prescribing pattern based on region, with an almost equal proportion of surgeons in Asia, South America and North America. There was no statistically significant difference in prescribing pattern based on years in practice, or training (Table 2).

**Table 2:** Respondent characteristics in prescribing standard physical therapy

	Standard physical therapy		p-value
	No	Yes	
Total	196	67	
Region			0.591
North America	132	43	
Asia	29	8	
South America	7	2	
Europe	23	13	
Middle East	5	1	
Years in Training			0.272
0 to 10	54	15	
11 to 20	55	26	
21 or more	85	26	
Training			0.200
Orthopedic Spine Surgeon	113	46	
Pediatric Orthopedic Surgeon	74	21	
Neurosurgical Spine Surgeon	2	0	
Other	7	0	

Among the 67 surgeons who prescribe standard PT, the most common indication was the use of standard PT in conjunction with brace treatment (58 %), small curves (48 %) or post-operatively (Table 3). Only 25 % of surgeons prescribed PT for treatment of pain. However, alleviation of pain was chosen as the most common goal of therapy (72 %). Improving aesthetics (40 %) and improving post-operative outcomes (37 %) were the next most common goals of standard PT. Of the 196 (75 %) surgeons who do not refer AIS patients for standard PT, the majority (149, 76 %) stated the lack of supporting research as the most common reason, followed by the perception that standard PT had no value in the treatment of AIS (112, 57 %).

**Table 3:** Stated indications and goals for standard physical therapy (*N* = 67)

Indication	Frequency	Percentage
Small curves	32	48 %
Braced Curves	39	58 %
Pre-operative	17	25 %
Post-operative	25	37 %
Patient request	7	10 %
Pain	17	25 %
Goals		
Prevent Progression	12	18 %
Improve Aesthetics	27	40 %
Treat Pain	48	72 %
Treat Respiratory Symptoms	11	16 %
Prevent Surgery	6	9 %
Postpone Surgery	3	4 %
Improve Post operative Outcomes	25	37 %
Maintain Improve Core Strength	7	10 %
Exercise with Bracing	1	1 %
Improved quality of life	1	1 %
Maintain flexibility	1	1 %
To get back to activities	1	1 %
Why don’t you prescribe Standard Physical Therapy?		
No access	8	4 %
No Patient Interest	20	10 %
No value	112	57 %
No research	149	76 %

Fifty-eight (22 %) surgeons reported referring their AIS patients for PSSE. There was no statistically significant difference in referral pattern based on region or surgical training. Surgeons in practice for ten years or less were less likely to refer for PSSE compared to surgeons who have been in practice for a longer period of time (Table 4).

**Table 4:** Respondent characteristics in prescribing Scoliosis specific exercises

	Scoliosis specific exercises		*p*-value
	No	Yes	
Total	204	58	
Region			0.520
North America	140	34	
Asia	25	12	
South America	7	2	
Europe	27	9	
Middle East	5	1	
Years in Training			0.022
0 to 10	62	7	
11 to 20	60	21	
21 or more	81	29	
Training			0.824
Orthopedic Spine Surgeon	124	35	
Pediatric Orthopedic Surgeon	72	22	
Neurosurgical Spine Surgeon	2	0	
Other	6	1	

Among the 58 surgeons who refer patients for PSSE, the most common indication was similar to standard PT: in conjunction with brace treatment (84 %) or for small curves (72 %) (Table 5). However, unlike standard PT, PSSE was less often prescribed for treatment of pain (3 %). Consistent with the respondents indication for referral, stated goals for PSSE were most commonly to improve aesthetics (62 %), to prevent curve progression (60 %) and to improve quality of life (53 %). The most common specific PSSE used was Schroth (33, 57 %), followed by Side Shift (13, 22 %), SEAS (12, 21 %) and FITS (11, 19 %) with almost similar distribution (Table 5). For the 204 (78 %) of surgeons who do not refer for PSSE, the main reasons were similar to standard PT, the lack of supporting research (149, 73 %), followed by the perception that PSSE had no value (108, 53 %). In addition, 63 (31 %) reported that they had no access to a facility providing PSSE.

**Table 5:** Stated type, indications and goals of Scoliosis specific exercise (*N* = 58)

Scoliosis specific exercise	Frequency	Percentage
Schroth	33	57 %
SEAS	12	21 %
FITS	11	19 %
Side Shift	13	22 %
Lyon	4	7 %
Dobomed	1	2 %
Indications		
Small curves	42	72 %
Braced Curves	49	84 %
Pre-operative	18	31 %
Post-operative	12	21 %
Patient request	3	5 %
Pain	2	3 %
Non-athletes in particular	1	2 %
Goals		
Prevent Progression	35	60 %
Prevent Surgery	19	33 %
Postpone Surgery	12	21 %
Improve Post-operative Outcomes	20	34 %
Improve Aesthetics	36	62 %
Improve quality of life	31	53 %
Decrease pain when present	1	2 %
General Fitness	1	2 %
Improve brace results	1	2 %
Muscle strengthening	2	3 %
Why don’t you prescribe Scoliosis Specific Exercise		
No access	63	31 %
No Patient Interest	12	6 %
No value	108	53 %
No research	149	73 %
Cost	4	2 %
Lack of exposure/experience	5	2 %

Slightly more than half of the surgeons (140, 53 %) noted an increased interest in PSSE, based mostly from inquiries from patients and their families (Table 6). When asked what factors might increase the role of PSSE in the treatment of AIS, most surgeons stated increased evidence of efficacy. Perhaps, due to this identified need, a large majority of surgeons (223, 86 %) favored funding PSSE studies by the SRS.

**Table 6:** Responses to “Have you observed an increased interest in SSE?”

Yes	140
Patients ask	113
More referrals	18
More research/publications	39
Hospitals promoting	20
No	123
No patient interest	49
No physician education	50
No research	84
Not offered	51

## Discussion

Treatment effectiveness of nonsurgical interventions for AIS remains under studied [18–20], with contradictory recommendations from research studies [2, 8, 20] and insufficient evidence to guide treatment. Specifically, Physiotherapeutic Scoliosis Specific Exercise (PSSE) treatment for AIS, although increasingly prevalent in Europe [1], have not been considered effective in North America. Spinal deformity surgeons, the Scoliosis Research Society and the Society on Scoliosis Orthopaedic and Rehabilitation and Treatment (SOSORT) [20] have recognized the lack of evidence supporting exercise treatment. The SRS funded a pilot study of PSSE, which resulted in a current RCT ongoing in Canada comparing standard PT to PSSE in adolescent scoliosis patients at a single center [21]. Despite a steady increase in the discussion surrounding PSSE, the view of PSSE among spinal deformity surgeons remains largely unknown. This study reports on survey results from 263 spinal deformity surgeons, relating to their opinions and attitudes concerning exercise treatment for Adolescent Idiopathic Scoliosis (AIS).

Reflective of the lack of strong evidence to support PT or PSSE to treat AIS, the majority (63 %) of respondents prescribed neither. Of those prescribing PT or PSSE, the number prescribing either or both was similar. In addition, despite the impression that PT and PSSE are more widely used in Europe [7–9, 15, 19, 20, 22] compared to North America, our study showed that there was no difference in prescribing patterns among the respondents based on region. Although, there was no difference in prescribing pattern for standard PT based on the surgeon’s years in practice, older surgeons were more likely to prescribe PSSE. PSSEs were developed in an era where clinical traditions and methods were passed on [23], unlike today where knowledge is spread through rigorous study design and publication of findings. Thus, surgeons who have been in practice longer may have had more exposure to PSSEs.

Consistent with current clinical indications, the majority of the respondents prescribe PT and PSSE for small curves [7, 20, 22] and as an adjunct to brace treatment [14–16]. Current guidelines recommend PSSE in curves less than 25° to avoid bracing by stabilizing the spine, and obtaining 3D autocorrection of the spine, pelvis and rib cage [20]. Twenty-five degrees is the generally accepted threshold at which brace treatment is recommended [20, 24] Although 25 % of surgeons prescribed PT for treatment of pain; alleviation of pain was chosen as the most common goal of therapy, and only 3 % prescribed PSSE for pain. This may illustrate some confusion regarding the benefits of exercise in the AIS population.

Despite an increased patient awareness of nonsurgical treatments for AIS, the majority of surgeons do not prescribe PT or PSSE. Respondents stated that they did not refer AIS patients for standard PT or PSSE as they felt that these treatments were of no value in AIS and that there was no supporting research. Recognizing this need, there was an overwhelming response that there was a need for better evidence demonstrating efficacy and most favored funding PSSE research using SRS research dollars.

There are limitations to this study, foremost of which is the relatively small number of respondents at 22 %. This may raise the possibility of selection bias in that only surgeons with a specific interest in the non-operative treatment of AIS may have responded. Yet a substantial number of respondents indicated that they do not prescribe either PT or PSSE. Still, this is a starting point in initiating the discussion on the need for a more robust study on the effectiveness of either PT or PSSE in the treatment of AIS.

While effective nonsurgical treatment for AIS would represent an important benefit for many patients, demonstration of treatment efficacy has generally proven difficult. However, the success of the BRAiST study has created a renewed focus on the potential for other nonsurgical treatments, and an increased optimism that nonsurgical treatments may be effectively studied. The results of this survey demonstrate significant skepticism regarding the benefit of either standard PT or PSSE in the management of AIS, which is to be expected given the lack of evidence. On the other hand, the fact those spinal deformity surgeons indicated overwhelming support for SRS funded research suggests a belief that at least some patients might benefit from PSSE treatment. It certainly indicates a growing understanding among SRS surgeons that evidence development is the optimal way to answer these important questions.

The results of this survey show that 22 % of the respondents use PSSE for AIS, although skepticism remains regarding the benefit of PSSE for AIS. Support for SRS funded research suggests belief that there is potential benefit from PSSE and the best way to assess that potential is through evidence development.
